# Exertional syncope caused by acquired obstruction of the pulmonary artery due to an organized thrombus in a dog

**DOI:** 10.1016/j.vas.2026.100929

**Published:** 2026-11-06

**Authors:** V. Szatmári, W. Bergmann, G.C.M. Grinwis

**Affiliations:** a Department of Clinical Sciences, Faculty of Veterinary Medicine, Utrecht University, Yalelaan 108, 3584 CM, Utrecht, the Netherlands; b Department Biomolecular Health Sciences, Faculty of Veterinary Medicine, Utrecht University, 3584 CL Utrecht, the Netherlands

**Keywords:** Angiography, Boxer, Epilepsy, Exercise-induced collapse, Pulmonary artery dissection, Holter, Pulmonary thromboembolism, Pulmonic stenosis, Seizure

## Abstract

An 8.5-year-old Boxer was referred for evaluation of frequently occurring exercise-induced seizure-like episodes for 2.5 months. Cardiac auscultation revealed a left basilar systolic murmur with an intensity of IV/VI. Echocardiography showed severe pulmonary arterial obstruction with a pressure gradient of 90 mmHg due to a suspected intraluminal mass. Cardiac catheterization was performed, where a right ventriculogram revealed a large rounded filling defect in the pulmonary artery trunk. No balloon angioplasty, biopsy or stenting were attempted. After euthanasia, post-mortem examination revealed an organized thrombus of unknown etiology attached to the wall of the pulmonary artery trunk. The adjacent arterial wall showed a markedly thickened intimal layer. The etiology remains unclear, primarily due to the chronic nature of the condition. Antemortem blood tests and postmortem examination failed to reveal any prothrombotic conditions. Acquired causes of pulmonary arterial obstruction are extremely rare in dogs, and an organized thrombus has not been reported yet.

## Highlights

• Exertional syncopal episodes manifested as seizures in this dog.
• Echocardiography and angiography revealed a soft tissue mass in the pulmonary trunk.
• Organized thrombus can cause acquired obstruction of the pulmonary artery in dogs.

## Case description

An 8.5-year-old intact male Boxer was referred to the cardiology service at the authors’ institution for evaluation of exercise-induced collapse occurring over a 2.5-month period. The episodes, which lasted <1 min, occurred several times daily, with a total of 151 episodes reported, and were triggered by excitement or exercise, including normal walks. Typically, the dog initially developed a stiff gait before falling into lateral recumbency and losing consciousness, accompanied by opisthotonus, cycling movements of the forelimbs and to a less extent the hindlimbs, and often urination. After approximately 30 s, the dog stood up, and immediately regain normal neuromuscular function. Between episodes, the dog was clinically normal, with no respiratory signs such as coughing or dyspnea.

The dog was initially referred for a brain magnetic resonance imaging (MRI), which revealed no abnormalities. Epilepsy was suspected, but treatment with imepitoin, phenytoin, oral potassium bromide, and levetiracetam had no effect in reducing the collapsing episodes. The dog was subsequently referred to another center, where blood tests and Holter electrocardiographic (ECG) monitoring were performed. Hematology and serum biochemistry were unremarkable, apart from a mildly increased C-reactive protein concentration (11.4 mg/L, reference, <10.7 mg/L). Holter ECG showed, besides seven isolated premature ventricular complexes in 24 h, no abnormalities. During one syncopal episode sinus tachycardia (150 BPM) was noted, during another one sinus arrhythmia (120 BPM) was observed, and during the third and fourth episodes sinus bradycardia (43 BPM) was recorded. Theophylline administration was started, but because the treatment did not result in resolution of the syncopal episodes, the dog was referred for pacemaker implantation to the authors’ institution.

At presentation the dog was bright, alert and responsive, with a body condition score of 4/9 (28.5 kg), and costo-abdominal breathing pattern with 24 breaths/min. The femoral pulses were strong, symmetric and regular with a rate of 132 BPM. Rectal temperature was 38.0 degrees of Celsius. Mucous membranes were pink. Cardiac auscultation revealed a left basilar grade IV/VI systolic murmur. Echocardiography revealed a severe acquired pulmonary arterial obstruction with a Doppler-derived pressure gradient of about 90 mmHg (Fig. 1). Though the image quality was insufficient to assess the pulmonary artery in detail, an echogenic structure was suspected in the pulmonary artery trunk as the cause of obstruction (Fig. 2, Video 1). The remaining echocardiographic findings were flattening of the interventricular septum, severe eccentric and moderate concentric right ventricular hypertrophy, moderate tricuspid valve regurgitation (pressure gradient: 86 mmHg), right atrial dilation, mild underfilling of the left ventricle, diffusely thickened aortic valve leaflets with a mild aortic stenosis (Doppler-derived pressure gradient: 28 mmHg). No cavitary effusions were present.

**Fig. 1:**
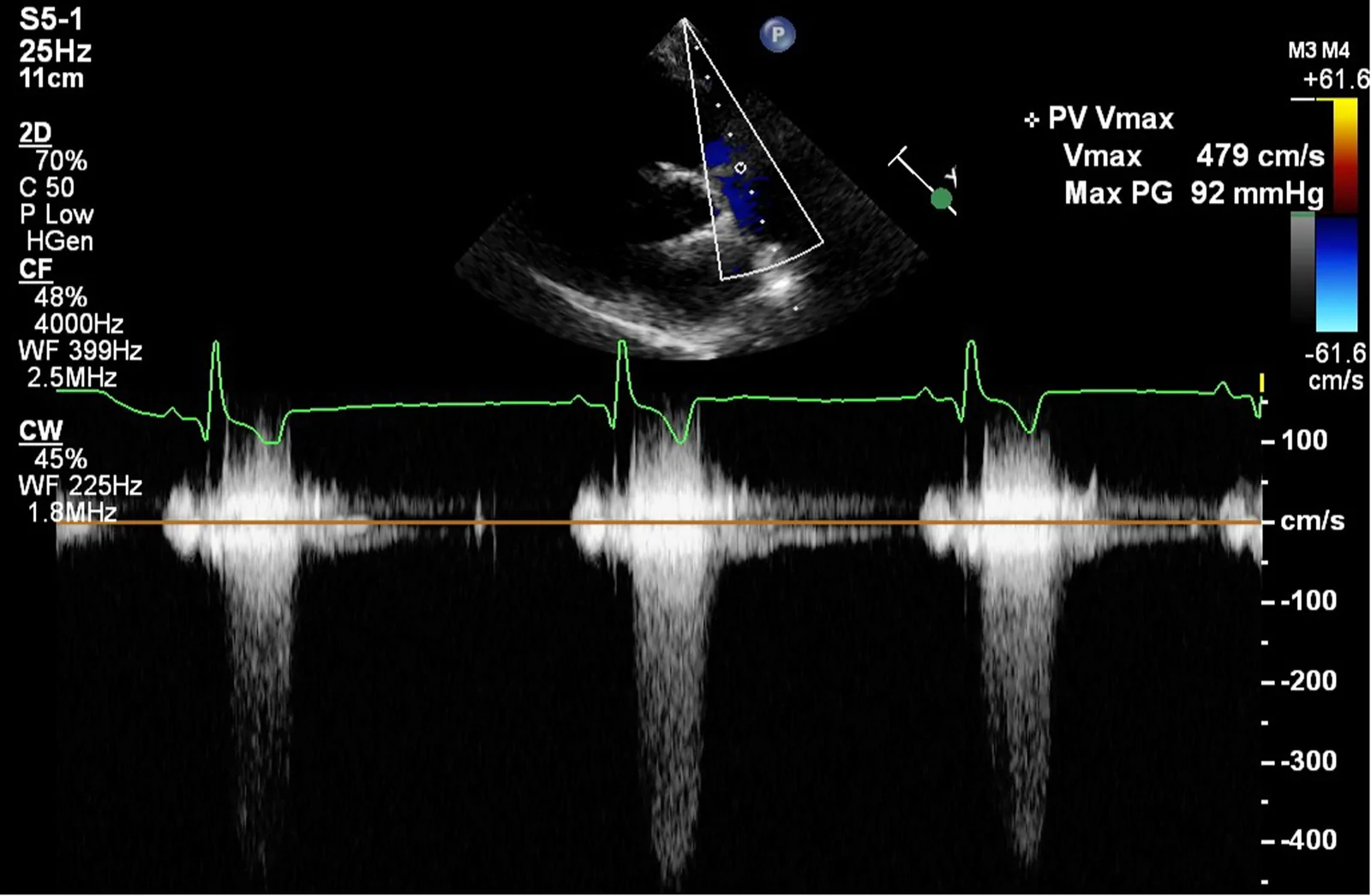
Continuous-wave Doppler echocardiography of the pulmonary trunk from left parasternal cranial standard image shows a severe pulmonary arterial obstruction with a pressure gradient of 92 mmHg. The focal indicator (o) on the continuous-wave Doppler cursor line is located at the level of the pulmonic valve.

**Fig. 2:**
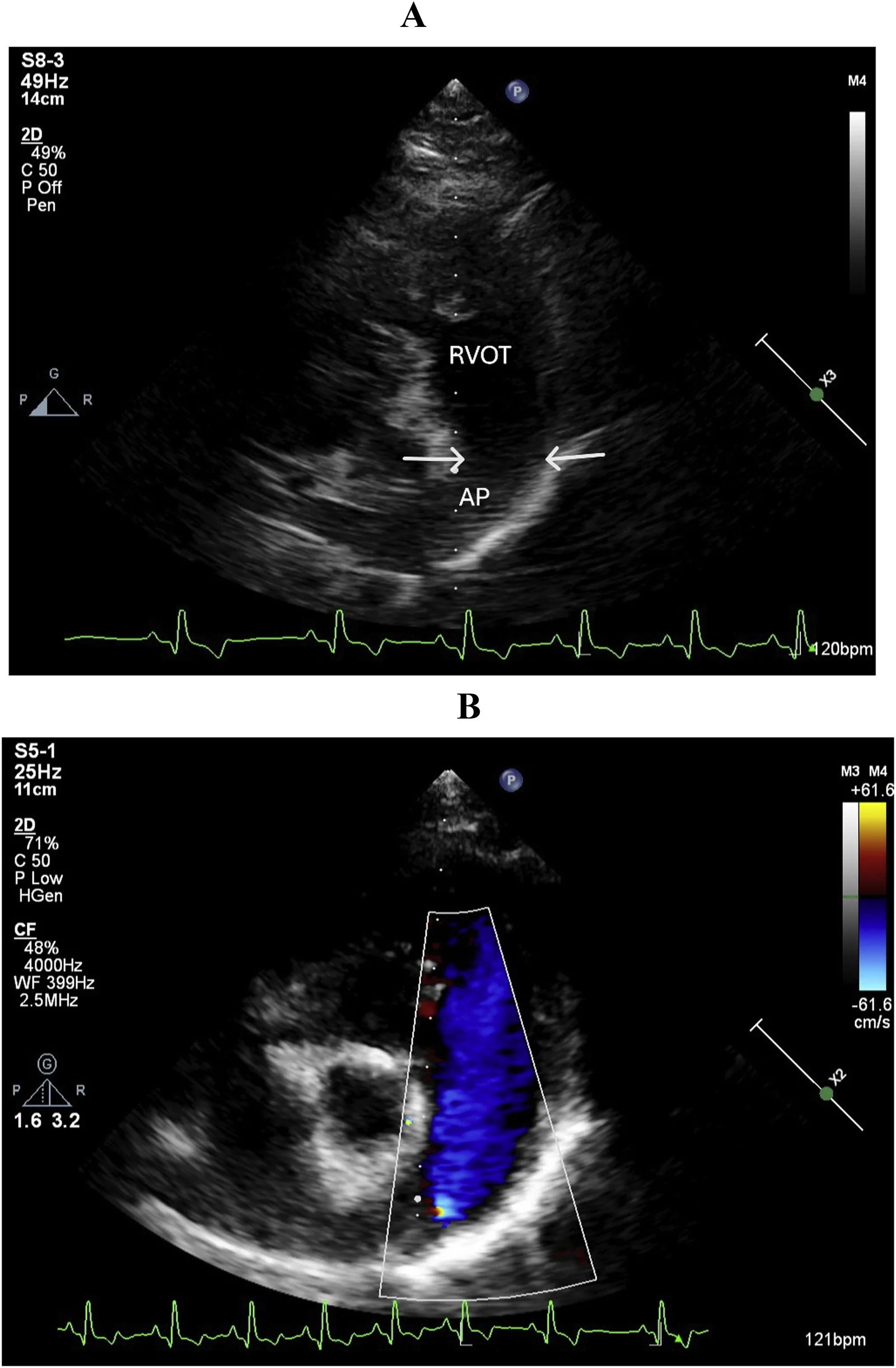
Echocardiography of the right ventricular outflow tract and pulmonary artery trunk from a standard right parasternal short-axis view. A. Two-dimensional image shows a mildly echogenic structure (distal to the arrows) distal to the pulmonic valve. The valve cannot be clearly visualized, but it should be at the level of the adjacent aorta, at about 4 o’clock location. RVOT = right ventricular outflow tract; AP = pulmonary artery trunk B and Video 1. Color Doppler mode shows laminar flow in the right ventricular outflow tract. At the level of the valve aliasing artifact (i.e., the mosaic color) and lack of flow can be appreciated suggesting an obstruction by a soft-tissue mass.

Additional blood tests were performed to investigate the possibility of thrombosis or thromboembolism. D-dimer concentration was slightly elevated (311 µg/l, reference <250 µg/l) and antithrombin concentration was within the reference range (119%, reference 89–146%). Antigen tests for *Dirofilaria immitis* and *Angiostrongylus vasorum* were negative.

The owner was offered a cardiac CT-scan or a cardiac catheterization to better understand the anatomy of the space occupying lesion in the pulmonary artery trunk. Cardiac catheterization was selected because it also offered the possibility of a potential therapeutic intervention during the same procedure.

Under general anesthesia, a routine right heart catheterization was performed with the dog in right lateral recumbency with vascular access via the right femoral vein (Scansen, 2023). A right ventriculogram revealed a large rounded filling defect in the pulmonary artery trunk (Fig. 3, Video 2). After the angiography, direct pressure measurements were performed using an end-hole multifunctional angiographic catheter. Right ventricular pressures were 58/9 mmHg. Distal to the filling defect, the pulmonary artery pressures were 22/8 mmHg; which resulted in a peak-to-peak systolic pressure gradient of 36 mmHg.

**Fig. 3:**
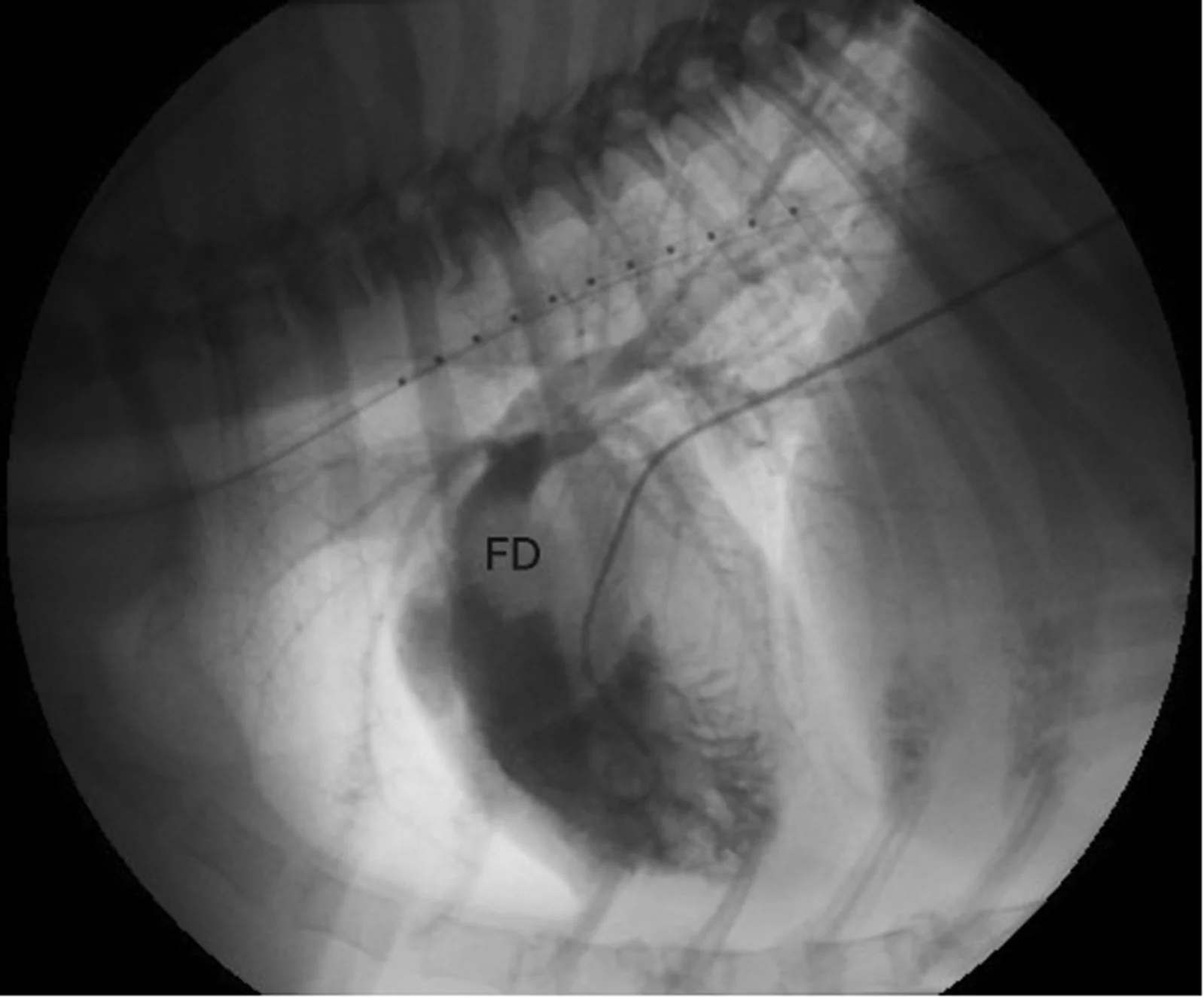
and Video 2 Right ventriculogram with power injection of 30 ml iodinated contrast material (1 ml/kg) via an angled 7 French pigtail catheter shows a large circular filling defect (FD) of about 3 by 2 cm at the level of the pulmonary trunk, distal to the pulmonic valves. Only a small volume of contrast material is able to pass at the cranial aspect of the artery. The filling defect noted on angiography is consistent with an intraluminal or mural mass, causing severe pulmonary arterial obstruction. There is a sizing catheter in the esophagus with 10 mm increments.

Because of the uncertain prognosis after the angiographic findings, the owner elected for immediate euthanasia, and gave consent for post-mortem examination.

Necropsy confirmed the echocardiographically detected right ventricular concentric and eccentric hypertrophy, a soft tissue mass in the pulmonary artery trunk, a right atrial dilation, and the slightly thickened aortic valve leaflets. In the pulmonary artery trunk a firm mass of about 3 × 2 × 1 cm was found, attached to the vessel’s wall, protruded into the lumen, just distal to the pulmonary valve, extending to the bifurcation (Fig. 4). The pulmonic valves looked macroscopically normal.

**Fig. 4:**
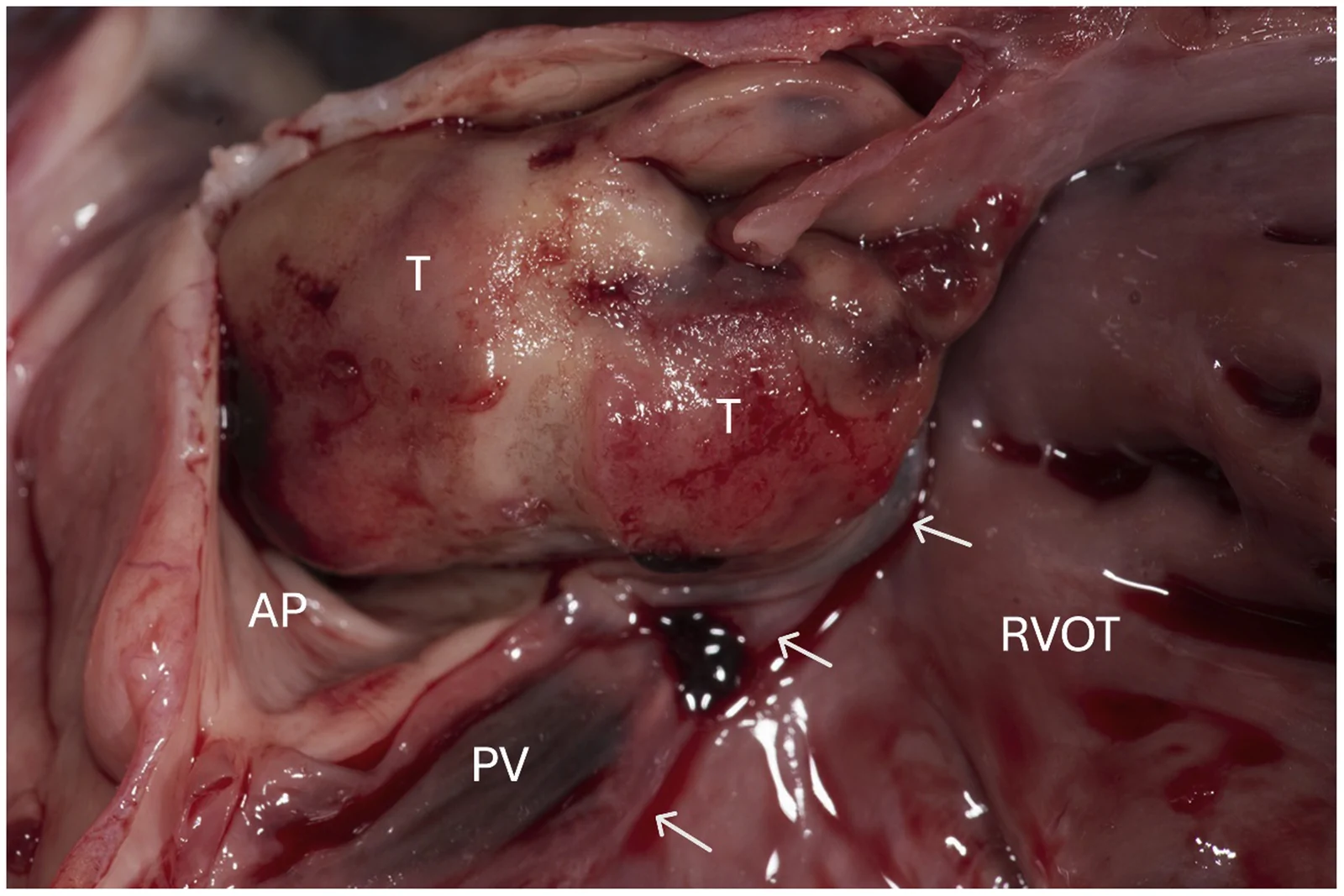
A pale, firm mass (T) is visible immediately distal to the pulmonary valve (PV) in the lumen of the pulmonary artery trunk (AP). The arrows indicate the pulmonary valve annulus, RVOT = right ventricular outflow tract.

Histologically, the intraluminal mass consisted of a fibrin core, with in the periphery, moderate numbers of laminar organized, neutrophil granulocytes and macrophages, partly covered by endothelium (Fig. 5). The base of the mass consisted of fibrotic tissue with moderate numbers of hemosiderophages and few lymphocytes, consistent with an organized thrombus. The adjacent wall of the pulmonary artery showed a markedly thickened intimal layer, compatible with arteriosclerosis. No arteriosclerotic changes were found in the other parts of the pulmonary artery trunk or any other vessels. Post-mortem examination revealed no inflammatory or neoplastic processes in any organ.

**Fig. 5:**
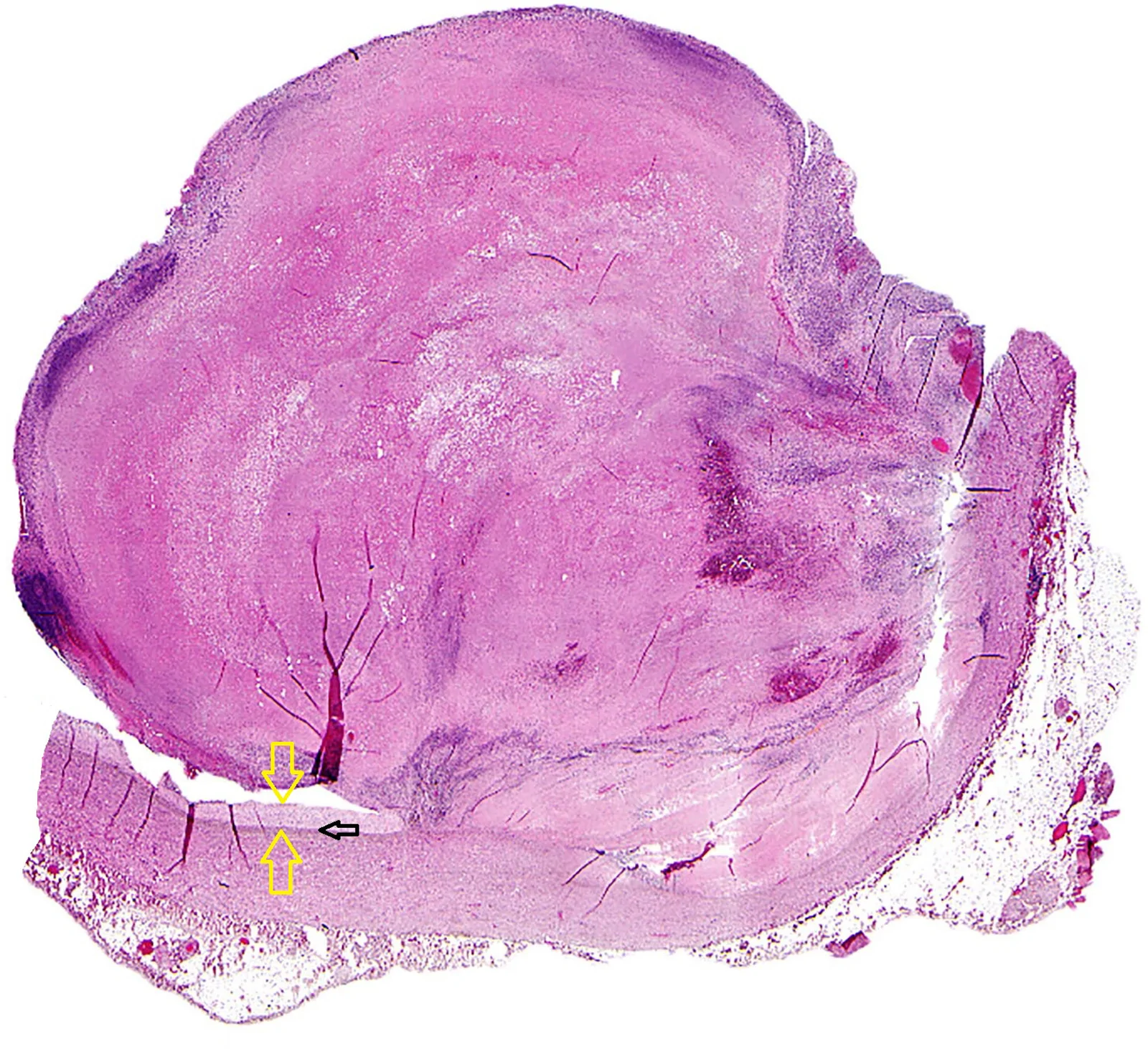
Photomicrograph of the intraluminal pulmonary artery mass at the attachment site to the vessel wall. The core of the mass is characterized by abundant granular/amorphous hypereosinophilic material (compatible with fibrin), cells with hypereosinophilic cytoplasm without nucleus (coagulative necrosis), a moderate amount of cellular debris, and karyorrhectic nuclei. This is surrounded by concentric layers of fibrin, moderate hemorrhages, fibrotic tissue, and moderate amount of neutrophil granulocytes and macrophages. The mass is compatible with a chronic organized thrombus. The vessel wall adjacent to the mass shows a markedly thickened intimal layer (between the yellow arrows) by intimal/subintimal fibrosis, compatible with arteriosclerosis. The true intima is indicated with the black arrow. Hematoxylin and eosin (HE) staining.

## Discussion

Congenital pulmonary artery valve stenosis is one of the most common congenital heart diseases in dogs, and exertional syncope is a commonly occurring clinical sign (Scansen, 2023). However, acquired causes of pulmonary arterial obstruction in dogs are rare, and can be caused either by an intraluminal obstruction or an external compression (Fox et al., 1984; Grint & Kellihan, 2017; Mikulak et al., 2022; Scansen et al., 2008; Scansen et al., 2015). Intraluminal and extraluminal neoplasia, as well as pulmonary artery dissection have been described in a handful of case reports in dogs (Fox et al., 1984; Grint & Kellihan, 2017; Mikulak et al., 2022; Scansen et al., 2008; Scansen et al., 2015). The present report describes a novel, acquired cause of pulmonary arterial obstruction, an organized, presumed *de novo* (i.e., in situ) thrombus. Based on the histopathologic findings, the large amount of amorphous eosinophilic material in the mass is compatible with fibrin, therefore it was diagnosed as a thrombus. The thrombus was described as organized because it contained fibrotic tissue arising from the vessel’s wall, as well as macrophages and neutrophil granulocytes. When a thrombus cannot be dissolved, organization and recanalization will take place. Endothelial cells from the healthy parts of the vessel’s wall migrate across the surface of the thrombus, wrapping it so that it is no longer directly exposed to the blood. Macrophages and neutrophilic granulocytes arising from the vessel’s wall and surrounding blood will invade the clot to release proteolytic enzymes to digest the fibrin, trapped red blood cells, and cellular debris. Also fibroblasts and smooth muscle cells migrate from the tunica media and adventitia of the blood vessel’s wall into the thrombus. These cells lay down an extracellular matrix, rich in collagen and proteoglycans, converting the soft clot into a firm, organized mass of fibrous connective tissue. Finally, capillaries sprout from the vessel’s wall and bore into the organizing thrombus. Over time, these newly formed microvascular channels can fuse together to form continuous passages through the blockage. This recanalization process can partially restore blood flow.

Possible etiologies of an organized thrombus at this location can be (1) a *de novo* (i.e., in situ) formed thrombus, (2) a thrombo-embolus, (3) a pulmonary artery dissection, or (4) an intramural hematoma (Kang et al., 2012; Perrotta & Lentini, 2015; Visser & Johnson, 2026). Based on the histological findings, a *de novo* thrombus is the most likely etiology; however, this cannot be proved with certainty, mainly because of the chronicity of the process. The difference between a *de novo* thrombus and a thromboembolism is that a thrombus is formed at that same location of the vascular system where it causes the obstruction, while a thromboembolus is formed elsewhere, proximal to the site of obstruction, either in the venous system or in the heart. After dislodgement, the thrombus becomes a thrombo-embolus, and it is carried by the blood flow to the pulmonary vascular tree, where it gets lodged, and subsequently causes an obstruction (Cao et al., 2021; Goggs et al., 2009; Kang et al., 2012; Liu et al., 2020; Matusov & Tapson, 2021; Perrotta & Lentini, 2015; Visser & Johnson, 2026). For a thrombus-formation, Virchow’s triad is a prerequisite, which consists of (1) endothelial damage, (2) stasis of the blood and (3) hypercoagulable state (Cao et al., 2021; Goggs et al., 2009; Kang et al., 2012; Liu et al., 2020; Matusov & Tapson, 2021; Perrotta & Lentini, 2015; Visser & Johnson, 2026) Because no underlying prothrombotic condition, such as inflammation, infection, neoplasia, hemolytic anemia, hypercortisolism or albumin loss, was identified, the underlying cause of thrombus-formation is hard to explain in the present case. However, it remains possible that the dog had a previous prothrombotic state, even if active thrombosis was no longer present at the time of diagnosis. It is unknown whether the focal arteriosclerotic changes of the pulmonary artery vessel wall adjacent to the thrombus were present before the thrombus formation, and with that it contributed to the pathogenesis of thrombus-formation, or these changes are the consequences of the turbulent blood flow that was caused by the mass. The reason why a pulmonary thromboembolism is considered a less likely etiology in the present case is that an embolus would get lodged in a smaller pulmonary artery branch or at a bifurcation (i.e., a saddle pulmonary thromboembolism). In addition, it would subsequently lead to pulmonary hypertension and probably also to an acute dyspnea as a leading clinical sign, resulting from ventilation-perfusion mismatch (Goggs et al., 2009). In the present dog no dyspnea was noticed.

Dissection of the pulmonary artery is caused by a mechanical injury to the inner layer of the vessel, allowing blood from the lumen to flow between the layers of the vessel wall, forcing the layers apart (Grint & Kellihan, 2017; Mikulak et al., 2022; Scansen et al., 2015). Pulmonary artery dissection has been reported after interventional catheterization procedures in dogs (Grint & Kellihan, 2017; Mikulak et al., 2022; Scansen et al., 2015), but the present dog had not undergone one. In humans, pulmonary hypertension is another known condition that can lead to a dissection, but the presented dog did not have pulmonary hypertension either (Cao et al., 2021; Kang et al., 2012; Liu et al., 2020; Matusov & Tapson, 2021; Perrotta & Lentini, 2015; Visser & Johnson, 2026). False lumen is a characteristic pathological finding of dissection, which was not found in our case. In addition, in cases of dissection a separation of the inner and outer media layer of the vessel’s wall can be appreciated on histology (Cao et al., 2021; Kang et al., 2012; Liu et al., 2020; Matusov & Tapson, 2021; Perrotta & Lentini, 2015).

Intramural hematoma has been described in humans as a rare cause of thrombus-formation in the wall of large vessels, among others in the pulmonary artery (Kang et al., 2012). An intramural hematoma is caused by a hemorrhage from a damaged intramural vessel. In this case, the thrombus would be completely covered by endothelial cells. The fact that the surface of the thrombus was only partially covered by endothelial cells in our case was more compatible with neoendothelization, which makes an intramural hematoma less likely (Cao et al., 2021; Kang et al., 2012; Liu et al., 2020; Matusov & Tapson, 2021; Perrotta & Lentini, 2015).

The clinical presentation of the described case, particularly the seizure-like episodes suggested the referring veterinarian an epilepsy. Differential diagnosis would consists of a primary cerebral pathology (such as neoplasia at this age), or a metabolic disorder (such as hypoglycemia). However, the presence of a loud cardiac murmur, and the fact that the syncopal episodes occurred during exercise, should raise the suspicion of an underlying cardiac problem, specifically a severe pulmonic or aortic stenosis with the left basal point of maximal intensity, or a severe pulmonary hypertension. The left basal localization of the murmur makes pulmonary hypertension unlikely, as this would lead either to no murmur or a right-sided systolic murmur secondary to a tricuspid valve regurgitation (Goggs et al., 2009; Visser & Johnson, 2026).

Antemortem diagnosis of the organized thrombus in the present case might have been possible with a cardiac computed tomography scan, and/or a histologic biopsy. Measuring D-dimer concentrations in combination with a CT-scan are often used in dogs with suspected pulmonary thromboembolism. In the present case, D-dimer concentration was mildly elevated. Plasma D-dimer levels in dogs have a reported sensitivity of 80% in diagnosing pulmonary thromboembolism (Epstein et al., 2013; Jeffrey et al., 2016). In our case, the D-dimer concentration was only mildly increased, and potentially lower than might have been expected given the presence of a relatively large thrombus. There are a number of possible reasons for this finding. The D-dimer levels are elevated with active fibrinolysis; however, if a blood clot is old, as suspected in this dog, active lysis drops markedly, resulting in lower circulating D-dimer levels. Another possible reason for lower than expected D-dimer levels can be a relatively small thrombus, which releases a small quantity of degradation products that might become diluted, even below the detection threshold. In the present case this latter scenario seems unlikely, as the thrombus was quite large. In addition, the used assay, and the laboratory’s reference ranges might influence the diagnostic performance of D-dimers (Brown et al., 2024; Török-Nagy et al., 2020). Specificity of plasma D-dimer levels is even lower than its sensitivity for detecting a thrombus in dogs (Brown et al., 2024; Epstein et al., 2013; Jeffrey et al., 2016; Török-Nagy et al., 2020). Many physiologic and disease processes, among others neoplasia, which latter was a major imaging differential diagnosis in the present case, can lead to increased D-dimer plasma concentrations (Török-Nagy et al., 2020).

In conclusion, an organized thrombus should be considered as a differential diagnosis for a mass-like lesion causing acquired obstruction of the main pulmonary artery in dogs.

## Funding

This research received no external funding.

## Statement for studies in humans or animals

The present report involves a privately owned animal that was treated according to best clinical practice and informed owner consent.

## CRediT authorship contribution statement

**V. Szatmári:** Writing – original draft, Visualization, Supervision, Software, Resources, Project administration, Methodology, Investigation, Formal analysis, Data curation, Conceptualization. **W. Bergmann:** Writing – review & editing, Visualization, Investigation. **G.C.M. Grinwis:** Resources, Investigation.

## Declaration of competing interest

The authors declare that they have no known competing financial interests or personal relationships that could have appeared to influence the work reported in this paper.
